# Factors associated with preventive behaviors regarding Lyme disease in Canada and Switzerland: a comparative study

**DOI:** 10.1186/s12889-015-1539-2

**Published:** 2015-02-25

**Authors:** Cécile Aenishaenslin, Pascal Michel, André Ravel, Lise Gern, François Milord, Jean-Philippe Waaub, Denise Bélanger

**Affiliations:** Groupe de Recherche en Épidémiologie des Zoonoses et Santé Publique (GREZOSP), Pavillon de la santé publique, Faculté de médecine vétérinaire, Université de Montréal, CP 5000, Saint-Hyacinthe, Québec Canada; Laboratory for Foodborne Zoonoses, Public Health Agency of Canada, CP 5000, Saint-Hyacinthe, Québec Canada; Laboratoire d’Eco-Épidémiologie, Institut de Biologie, Université de Neuchâtel, 2000 Neuchâtel, Suisse; Institut national de santé publique du Québec, Montréal, Québec Canada; Groupe d’étude et de recherche en analyse de la décision (GERAD), Université du Québec à Montréal, Montréal, Québec Canada

**Keywords:** Lyme disease, Borreliosis, Preventive behaviors, Prevention, Ticks, Risk perception, Tick bites

## Abstract

**Background:**

Lyme disease (LD) is a vector-borne disease that is endemic in many temperate countries, including Switzerland, and is currently emerging in Canada. This study compares the importance of knowledge, exposure and risk perception for the adoption of individual preventive measures, within and between two different populations, one that has been living in a LD endemic region for several decades, the Neuchâtel canton in Switzerland, and another where the disease is currently emerging, the Montérégie region in the province of Québec, Canada.

**Methods:**

A web-based survey was carried out in both study regions (814 respondents) in 2012. Comparative analysis of the levels of adoption of individual preventive measures was performed and multivariable logistic regression analyses were used to test and compare how knowledge, exposure and risk perception were associated with the adoption of selected measures in both regions and globally.

**Results:**

In Montérégie, the proportion of reported adoption of five of the most commonly recommended preventive measures varied from 6% for ‘applying *acaricides* on one’s property’ to 49% for ‘wearing *protective clothing’*, and in Neuchâtel, proportions ranged from 6% (*acaricides*) to 77% for ‘checking for ticks (*tick check)’*. Differences were found within gender, age groups and exposure status in both regions. The perceived efficacy of a given measure was the strongest factor associated with the adoption of three specific preventive behaviors for both regions*: tick check, protective clothing* and *tick repellent*. Risk perception and a high level of knowledge about LD were also significantly associated with some of these specific behaviors, but varied by region.

**Conclusions:**

These results strongly suggest that social and contextual factors such as the epidemiological status of a region are important considerations to take into account when designing effective prevention campaigns for Lyme disease. It furthermore underlines the importance for public health authorities to better understand and monitor these factors in targeted populations in order to be able to implement preventive programs that are well adapted to a population and the epidemiological contexts therein.

**Electronic supplementary material:**

The online version of this article (doi:10.1186/s12889-015-1539-2) contains supplementary material, which is available to authorized users.

## Background

Lyme disease (LD) is a multisystemic tick-borne disease that is caused by the bacteria *Borrelia burgdorferi* and has been endemic for several decades in the United States and in Europe. Recognized as the most frequent vector-borne disease in many temperate countries, LD is emerging in Canada [1]. In the province of Québec, Canada, the first indigenous cases were reported in 2008 [2]. In 2012, 42 cases were reported in the province (incidence of 0,5 per 100 000 inhabitants), the vast majority having occurred in the Montérégie region, a region situated in the south east of the province near the US border [3]. In Switzerland, LD has been occurring for over three decades [4]. The incidence varies between cantons, and the Neuchâtel canton has an incidence which is above the Swiss national mean with last estimates ranging from 49 to 95 cases per 100 000 inhabitants [5,6]. There is no vaccine for LD currently available. The two main strategies promoted to prevent LD rely on decreasing the contact rate between infected ticks and humans by: 1) reducing the infected tick density in the environment via environmental preventive measures, and 2) promoting the adoption of individual preventive measures by educating and raising LD awareness in populations at risk.

Environmental preventive measures include actions aimed at reducing tick density in the environment, such as the application of acaricides or landscaping, as well as actions targeted at tick hosts such as treatment of deer or rodents with topical or oral acaricides, the exclusion of deer by fencing, vaccination of rodents, and other actions (environmental preventive measures against LD are reviewed in Piesman and Eisen [7]). Most of these environmental measures have been demonstrated to reduce tick densities in experimental settings in a North American context, but not all of them have been demonstrated to reduce the risk of LD in populations. It should be noted however, that the ecology of LD differs between North America and Europe [8], and it has yet to be shown whether or not environmental measures are transferable from one ecological context to another, such as Switzerland and its neighboring countries. Given these circumstances, the main public health strategy adopted by most countries until now has focused on the promotion of preventive measures among populations at risk [7].

Individual preventive measures have demonstrated efficacy in preventing LD in populations [7,9-14]. The primary actions recommended in the group of individual preventive measures include wearing long trousers or putting one’s socks into one’s trousers when visiting wooded areas, applying tick repellent on skin and clothing, checking for and removing ticks after visiting wooded areas, and avoiding tick habitats during high risk periods [14]. The application of acaricides on one’s property has also been recommended in the US for those living in high risk regions [15].

Factors influencing the adoption of preventive behaviors have been extensively studied for many diseases and health issues using different theoretical models, one of the most widely used being the Health Belief Model [16]. They include demographic factors, accessibility of health care services, knowledge about the disease, risk perception of the disease, perceptions of the efficacy of the measure, and social network characteristics [17]. The four lasts factors are social cognitive factors (knowledge, attitudes, beliefs), and are of particular interest for public health authorities given that they constitute the determinants of preventive behaviors that are believed to be the most open to change in a population by means of communication campaigns [13,18]. Previous studies have looked at the determinants of preventive behaviors for LD in the United States [19-30] and in Europe [31-34]. All of these studies have focused on one country, and the general observation coming out of this body of research is that the proportion of the population that adopts preventive behaviors, as well as the importance of the determinants of adoption, varies by context, but most give no indication on how context influences these parameters. Only a few studies have formally compared the determinants of preventive behaviors between populations living in regions with different LD incidence [19,20], and none has studied the determinants of preventive behaviors in a region with emerging LD, nor has any study compared the differences between countries with LD endemic and emerging statuses. We believe that a better understanding of the relationship between the adoption of preventive measures and their determinants in different epidemiological situations represents a critical aspect of the design of targeted and effective preventive communication programs. Aenishaenslin and colleagues [35] have shown that the perceived susceptibility toward LD, one recognized determinant of the adoption of preventive behaviors, was considerably higher in the Montérégie region, a region where LD is emerging. Building on this previous work, we hypothesized that risk perception may have a stronger effect on the adoption of preventive measures for LD in an emerging context, in contrast to a region where LD is endemic, such as the Neuchâtel region.

The aim of this paper is to compare the adoption of preventive behaviors by individuals, as well as the relative importance of knowledge, level of exposure, risk perception and the perceived efficacy of preventive behaviors as potential determinants of such behaviors, within and between populations living in two different regions, one that has had endemic LD for the last 30 years, the Neuchâtel canton, in Switzerland, and another where the disease is currently emerging, the Montérégie region, in Québec, Canada. Currently, preventive actions toward LD in these two regions focus on risk communication.

## Methods

### Data collection

This cross-sectional study used data from web-surveys conducted simultaneously in fall 2012 in both study regions, the Montérégie region (n = 401) and the Neuchâtel region (n = 413). Details on the survey design and on data collection strategies are described in Aenishaenslin et al. [35]. The complete questionnaire in French is available in Additional file 1. This paper focuses specifically on assessing the level of adoption of five individual preventive measures in our two studied populations, namely: checking for ticks after outdoor activities (*tick check)*, wearing *protective clothing*, applying *tick repellent*, avoiding wooded areas during high-risk periods (*risk area avoidance*), and treating properties with *acaricides*. Survey construction was based on the Health Belief Model [16] and questions where designed to measure levels of adoption of specific individual preventive measures (“How often do you apply ‘this measure’ to protect yourself against LD?”: (0) never, (1) rarely, (2) often, (3) always), as well as user’s perceived efficacy of the measures, using a five point Likert scale (“this measure is effective for the prevention of LD”: (5) strongly agree, (4) somewhat agree, (3) neither agree or disagree, (2) somewhat disagree, (1) strongly disagree). For questions measuring behavior adoption, respondents could select ‘Does not apply to my situation’ and were then excluded from further analysis specific to this measure. Additional data collected in the study included: gender, age group, education level, level of exposure through outdoor activities (10 or more outdoor activities in a LD risk region during the risk period per year), and level of knowledge of LD (high if 3 or 4 good answers or low if 0 to 2 good answers, based on four LD knowledge related questions regarding mode of transmission of the disease, early symptoms, treatment, and risk zones). The study protocol, including the complete questionnaire, was reviewed by the ethical committee for health research of the University of Montreal (Comité d’éthique de la santé, CERES) (certificate number 12-050-CERES-D), and the ethical certificate was approved by the Université de Neuchâtel.

### Data analysis

A global preventive behavior score (GPB) (three levels: null, moderate or high) was computed based on three major recommended preventive measures by public health authorities in both studied regions [36,37]: *tick check,* use of *protective clothing*, and *tick repellent*. The GPB score was ‘high’ if respondents had a score of 2 (often) or 3 (always) for at least two of these three preventive measures, it was ‘moderate’ if they had a score of 2 (often) or 3 (always) for one of these measures, and ‘null’ in every other case. These three levels of GPB scores were used for descriptive analyses.

A global risk perception score was also calculated based on the mean score of four observed perception variables: perceived severity, perceived individual susceptibility, perceived regional susceptibility and feelings of worry as described in Aenishaenslin et al. [35].

Descriptive and multivariable statistical analyses were performed using IBM SPSS Statistics 19. The proportion of reported behavior adoption for the five individual preventive measures were calculated separately by region for respondents who declared that they had heard about LD before the survey and excluding those who considered that the measure didn’t applied to their situation (considered as ‘LD familiar respondents’ in the paper) and for the total survey population (total population). Pearson Chi-square statistics were calculated to assess significant differences (p < 0.05) between groups (study regions, gender, age groups, exposure status).

In order to measure and compare the effect of exposure, knowledge, global risk perception, and perceived efficacy of individual measures on behavior adoption, twelve multivariable logistic regression models were built using the following dependent variables: 1) GPB score (models A in Table 1) and 2) the specific adoption score for each of the three main preventive measures (*tick check*, use of *protective clothing* and *tick repellent*) (models B, C, D in Table 1). Among these, eight models were region specific and four were overall models combining both regions. In the regression models, the GPB score was dichotomized (scores of ‘high’ or ‘moderate’ become ‘1’ or ‘good’ and scores ‘null’ become ‘0’ or ‘inadequate’), as were the specific adoption scores for *tick check*, use of *protective clothing*, and *tick repellent* (scores of ‘2’ or ‘3’ become ‘1’ and scores of ‘0’ or ‘1’ become ‘0’) in order to enable their use as dependent variables. Independent variables included in the models were 1) the exposure level (high *vs* low), 2) the global LD knowledge score (high *vs* low) [35], 3) the global risk perception score (considered as a continuous variable) [35], and 4) the perceived efficacy of specific measures (only in the specific models). Gender, age and education level were considered as potential confounders and forced in all models for comparison sake. Only respondents with previous knowledge of LD (prior to survey administration) were included in the multivariable analysis.

## Results

Table 2 shows the proportion of respondents reporting adoption of the five preventive behaviors of interest by region, gender, age group and exposure status. Within LD familiar respondents, proportions in Montérégie varied from 6% for *acaricides* to 49% for use of *protective clothing* and fell to 3% (*acaricides*) and 22% (*protective clothing*) when considering the total Montérégie studied population. In Neuchâtel, proportions ranged from 6% for *acaricides* to 77% for *tick check* within LD familiar respondents, and from 3% (*acaricides*) to 57% (*tick check*) in the total studied population. Proportions for *tick check* and *protective clothing* adoption (73% for each) were higher in Neuchâtel (p < .0001) compared to Montérégie (18% and 49%, respectively) (Table 2).

**Table 1:** **Factors associated with general preventive behavior score (GPB) and with three specific preventive behaviors against LD**

**A) Factors associated with GPB** ^**1**^						
	**Montérégie (n = 201)**		**Neuchâtel (n = 312)**		**Overall model (n = 513)**	
	OR	95% CI	OR	95% CI	OR	95% CI
Exposure	1.17	(0.56-2.44)	2.23	(1.12-4.43)*	1.67	(1.02-2.73)*
Knowledge of LD	2.07	(1.05-4.10)*	2.32	(1.17-4.59)*	2.29	(1.42-3.68)**
Risk perception	1.79	(1.15-2.79)*	1.32	(0.81-2.16)	1.54	(1.12-2.12)**
Region (Montérégie: ref)	-	-	-	-	0.33	(0.20-0.54)***
**B) Factors associated with “Performing tick check after outdoor activities”** ^**1**^						
	**Montérégie (n = 166)**		**Neuchâtel (n = 298)**		**Overall model (n = 464)**	
	OR	95% CI	OR	95% CI	OR	95% CI
Exposure	1.15	(0.42-3.16)	1.24	(0.67-2.30)	1.22	(0.73-2.02)
Knowledge of LD	1.18	(0.47-3.00)	2.45	(1.31-4.59)**	1.90	(1.16-3.13)**
Risk perception	2.00	(1.05-3.78)*	1.62	(1.03-2.54)*	1.66	(1.17-2.34)**
Perceived efficacy of the measure	3.17	(1.18-8.55)*	11.90	(4.53-31.31)***	6.87	(3.38-13.97)***
Region (Montérégie: ref)	-	-	-	-	.12	(0.07-0.21)***
**C) Factors associated with “Wearing protective clothing”** ^**1**^						
	**Montérégie (n = 176)**		**Neuchâtel (n = 293)**		**Overall model (n = 469)**	
	OR	95% CI	OR	95% CI	OR	95% CI
Age 18–34 yr	1.24	(0.35-4.36)	.41	(0.19-0.88)*	.46	(0.25-0.86)*
35-54 yr	.35	(0.17-0.73)**	.85	(0.41-1.75)	.61	(0.37-0.99)*
55+ yr^R^	1		1		1	
Exposure	1.05	(0.47-2.38)	1.35	(0.76-2.41)	1.21	(0.77-1.91)
Knowledge of LD	2.29	(1.08-4.84)*	1.51	(0.83-2.75)	1.98	(1.27-3.10)**
Risk perception	1.84	(1.13-3.01)*	1.13	(0.74-1.70)	1.35	(1.00-1.83)*
Perceived efficacy of the measure	7.99	(1.65-38.68)*	35.45	(4.35-288.53)**	14.76	(4.30-50.62)***
Region (Montérégie: ref)	-	-	-	-	.46	(0.28-0.76)**
**D) Factors associated with “Applying tick repellent”** ^**1**^						
	**Montérégie (n = 173)**		**Neuchâtel (n = 290)**		**Overall model (n = 463)**	
	OR	95% CI	OR	95% CI	OR	95% CI
Age 18–34 yr	5.06	(1.23-20.71)*	1.24	(0.60-2.59)	1.55	(0.84-2.86)
35-54 yr	1.17	(0.50-2.74)	2.52	(1.31-4.82)*	1.89	(1.16-3.09)*
55+ yr^R^	1		1		1	
Exposure	1.54	(0.60-3.98)	1.09	(0.63-1.88)	1.13	(0.72-1.78)
Knowledge of LD	2.12	(0.87-5.13)	.84	(0.47-1.49)	1.10	(0.69-1.75)
Risk perception	1.51	(0.82-2.80)	1.86	(1.23-2.82)**	1.68	(1.21-2.35)**
Perceived efficacy of the measure	17.66	(6.63-47.04)***	8.83	(3.74-20.86)***	10.77	(5.78-20.04)***
Region (Montérégie: ref)	-	-	-	-	1.92	(1.11-3.31)*

^1^Gender, age and education level were forced in all models as potential confounders. Related OR are shown only if statistically significant in the model.

^R^Reference categories.

*p < 0,05; **p < 0,01; ***p < 0,001.

**Table 2:** **Proportions of reported adoption of LD preventive behaviors by region, gender, age groups and level of exposure**

**Preventive measure**	**Gender**				**Age**						**Level of exposure**				**Total** ^**1**^			**Total region** ^**2**^
	**Women**		**Men**		**18-34 yr**		**35-54 yr**		**55+ yr**		**High**		**Low**					
	**%**	**n**	**%**	**n**	**%**	**n**	**%**	**n**	**%**	**n**	**%**	**n**	**%**	**n**	**%**	**n**	**P**	**%**
Performing tick check after outdoor activities																		
Montérégie	16%	13/81	20%	17/85	11%	2/19	15%	10/69	23%	18/78	20%	8/40	18%	22/126	18%	30/166	<0.0001	7%
Neuchâtel	82%	155/190	69%^*^	79/114	78%	62/80	79%	106/135	74%	66/89	80%	138/172	73%	96/132	77%	234/304		57%
Wearing protective clothing																		
Montérégie	50%	42/84	49%	45/92	63%	10/16	36%**	27/75	60%	50/85	49%	19/39	50%	68/137	49%	87/176	<0.0001	22%
Neuchâtel	76%	139/183	69%	79/115	61%^2^	47/77	76%	102/135	80%	69/86	78%	131/169	67%^***^	87/129	73%	218/298		53%
Applying tick repellent																		
Montérégie	40%	35/87	30%	26/87	61% ^**^	11/18	35%	24/69	30%	27/87	43%	17/40	33%	44/134	35%	61/174	ns	15%
Neuchâtel	44%	82/185	33%	37/111	34%	27/79	49%**	65/134	33%	27/83	42%	70/165	37%	49/131	40%	119/296		29%
Avoiding wooded areas during high-risk period																		
Montérégie	45%	39/86	26%^*^	23/90	30%	6/20	23%^**^	17/73	47%^**^	39/83	14%	6/42	42%^***^	56/134	35%	62/176	ns	15%
Neuchâtel	36%	67/184	37%	38/104	31%	23/74	35%	45/130	44%	37/84	28%	45/159	47%^***^	60/129	37%	105/288		25%
Treating properties with acaricides																		
Montérégie	10%	8/83	3%	3/89	0%	0/17	3%	2/70	11%**	9/85	5%	2/37	7%	9/135	6%	11/172	ns	3%
Neuchâtel	5%	6/116	7%	5/71	4%	2/52	7%	6/87	6%	3/48	7%	7/101	5%	4/86	6%	11/187		3%
Gobal preventive behavior score																		
Montérégie	58%	59/102	55%	55/100	57%	13/23	52%	42/81	60%	59/98	60%	27/45	55%	87/157	56%	114/202	<0.0001	28%
Neuchâtel	89%	175/197	82%	99/121	86%	71/83	86%	121/141	87%	82/94	91%	161/177	80%^***^	113/141	86%	274/318		66%

^1^includes only respondents who knew of LD before survey administration (Proportion of adoption in ‘LD familiar’ respondents).

^2^includes all respondents (Proportion of adoption in the total population; Montérégie: n = 401, Neuchâtel: n = 413).

*Significant (p < 0,05) difference when compared to women in the same region (Pearson Chi-square statistic).

**Significant (p < 0,05) difference when compared to other age groups in the same region (Pearson Chi-square statistic).

***Significant (p < 0,05) difference when compared to the high exposed group in the same region (Pearson Chi-square statistic).

In Montérégie, the proportion of *protective clothing* users was lower in the 35-54 yr old group (36%) compared to other age groups (18-34 yr = 63%; 55 + yr = 60%) (p < 0.05) (Table 2). For this same age group, it was also lower for *risk area avoidance* with 23% versus 30% in the 18-34 yr old group and 47% in the 55 + yr old group (p < 0.05). For *tick repellent*, the proportion was higher in the 18-34 yr old group (61%) versus 35% and 30% in the 35–54 and 55 + yr old groups (p < 0.05). The proportion for *acaricides* was very low in all age groups but higher in the 55 + yr old group for this region (11% *vs* 0% in 18-34 yr and *vs* 3% in the 35-54 yr old group) (p < 0.05).

In Neuchâtel, the proportion of adopters for the *tick check* behavior was lower in men than women (69% vs 82% in women) (p < 0.05) (Table 2). The proportion of *protective clothing* adopters was lower in the low-exposure group with 67% vs 78% in the high-exposure group (p < 0.05). For *tick repellent* adopters it was higher in the 35-54 yr old group with 49% versus 34% and 33% in the 18–34 and 55 + yr old groups respectively (p < 0.05).

**Figure 1:**
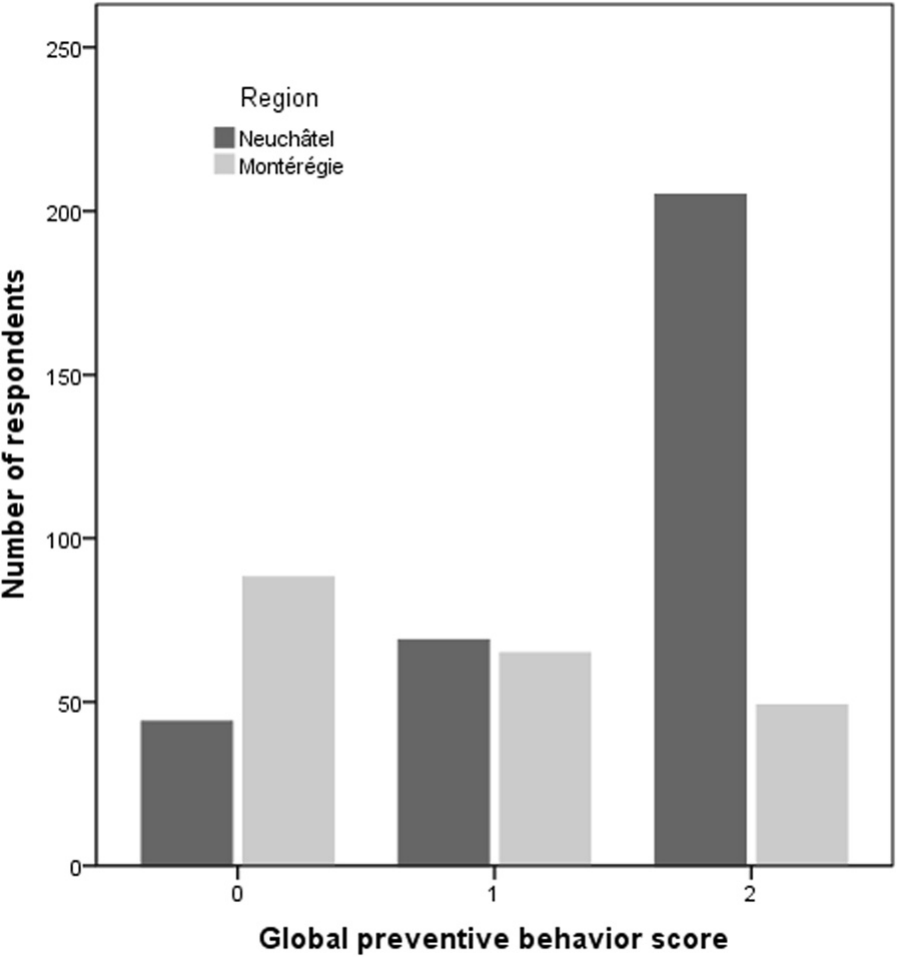
**Distribution of global preventive behavior scores (GPB) by region.**

With regards to the GPB score, 86% of the Neuchâtel respondents had a moderate (22%) or high score (64%), which was significantly higher than scores obtained in Montérégie where only 56% of respondents were found to have had moderate or high scores (Table 2, Figure 1). In Neuchâtel, the low exposure group had a lower proportion of moderate/high scores (80%) *vs* the high exposure group (91%) (p < 0.05) (Table 2).

**Figure 2:**
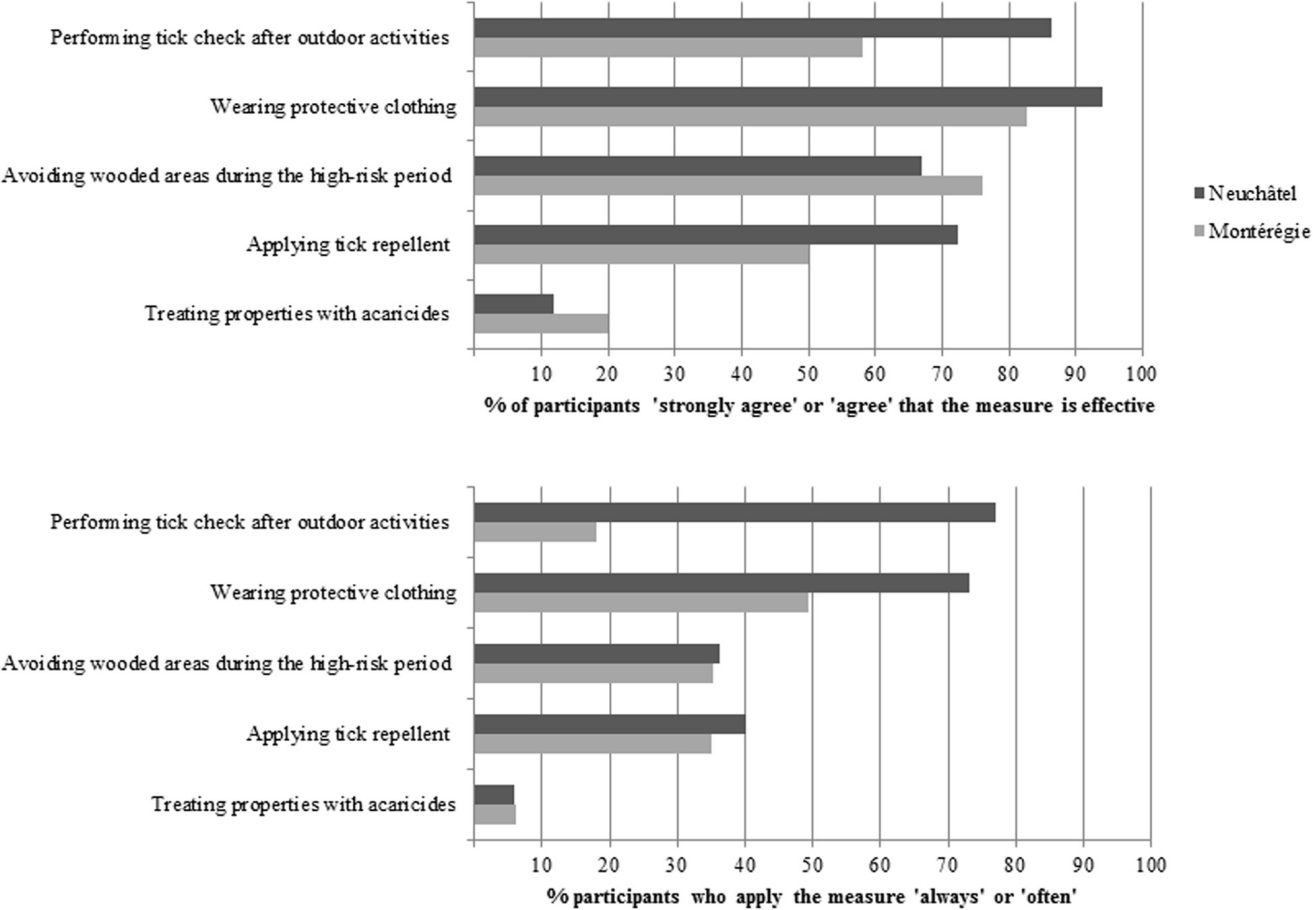
**Perceived efficacy and proportion of adoption of five preventive measures against Lyme disease by region.**

The perceived efficacy of the five measures varied between populations (Figure 2). In both populations, four measures were perceived as effective by at least 50% of the population, except for the use of *acaricides*, which was perceived as effective by only 12% in Neuchâtel and by 20% in Montérégie. In Neuchâtel, *protective clothing* (94%), *tick check* (86%) and *tick repellent* (67%) were the three measures most often perceived as effective, whereas in Montérégie, it was *protective clothing* (83%), *risk area avoidance* (76%) and *tick check* (58%) which were perceived as being the most effective. For all five measures, proportions were found to be different between regions (p < 0.005).

Table 1 presents the results from multivariable logistic regression analyses with four different dependent variables: (A) global preventive behavior score (GPB), (B) adoption of *tick check*, (C) adoption of *protective clothing* and (D) adoption of *tick repellent*, in regional subsets and in the overall sample.

With regards to the GPB score, high levels of knowledge (OR = 2.07, 95% CI = 1.05-4.10) and risk perception (OR = 1.79, 95% CI = 1.15-2.79) were both found to be significantly associated with a good GDP in Montérégie (good GDP is a moderate or high GDP, i.e. at least one of the three main preventive measures is reported as being adopted ‘often’ or ‘always’). In Neuchâtel, knowledge (OR = 2.32, 95% CI = 1.17-4.59) and exposure (OR = 2.23, 95% CI = 1.12-4.43) were found to be significant factors. In the overall model, knowledge (OR = 2.29, 95% CI = 1.42-3.68), exposure (OR = 1.67, 95% CI = 1.02-2.73), risk perception (OR = 1.54, 95% CI = 1.12-2.12) and region (OR = 0.33, 95% CI = 0.20-0.54) were significantly associated with good GPB.

When examining the six region specific models, we noted that significant factors varied by outcome. The perceived efficacy of specific measures was found to be significantly associated with the three studied behaviors in both regions with high odds ratios ranging from 3.17 (95% CI = 1.18-8.55) for *tick check* in Montérégie to 35.45 (95% CI = 4.35-288.53) for *protective clothing* in Neuchâtel. Risk perception was significantly associated with behaviors in four specific models*: tick check* (OR = 2.00, 95% CI = 1.05-3.78) and *protective clothing* (OR = 1.84, 95% CI = 1.13-3.01) in Montérégie and *tick check* (OR = 1.62, 95% CI = 1.03-2.54) and *tick repellent* (OR = 1.86, 95% CI = 1.23-2.82) in Neuchâtel. Knowledge was significantly associated with behaviors in two specific models: *tick check* in Neuchâtel (OR = 2.45, 95% CI = 1.31-4.59) and *protective clothing* in Montérégie (OR = 2.29, 95% CI = 1.08-4.84), whereas exposure was never significantly associated with specific behaviors in either region. In the overall specific models, perceived efficacy of the measure, risk perception and region were significantly associated with the three behaviors and knowledge with two of them: *tick check* and *protective clothing*. Age was associated with *protective clothing* and *tick repellent* in both regions. More precisely, being in the 35–54 years of age group was negatively associated with the use of *protective clothing* in Montérégie. In Neuchâtel, being in the 18–34 years of age group was also negatively associated with this behavior. Being in the 18–34 years of age group was positively associated with the use of *tick repellent* in Montérégie, and being in the 35–54 years of age group was positively associated with the adoption of this behavior in Neuchâtel.

Interactions between variables included were tested in each model but none were found to be statistically significant. Regarding comparisons of OR for the four dependent variables of interest, none were identified as different between regional subsets when considering the 95% confidence intervals in all models, other than the fact that different significant factors were identified in both populations.

## Discussion

One objective of this study was to compare the adoption of preventive measures by individuals within and between populations living in regions with different LD epidemiological statuses. Overall, in Neuchâtel, a high proportion of respondents (86% among those who knew of the disease before the survey) reported adoption of at least one of the main preventive measures and three out of four declared having checked for ticks after being in an area at risk for LD. With an incidence reaching 95 cases per 100 000 inhabitants [5], this is good news from a public health perspective given that removing ticks within 24 h after being bitten can reduce the risk of transmission of the bacteria to near zero [38,39]. This high level of adoption of preventive measures was not observed in the Montérégie region where the highest level of adoption for a preventive measure was found to be 50% for the use of *protective clothing*, among those who knew of LD before they had taken the survey, and under 20% for *tick check* and 35% for *tick repellent*. This finding may reflect a lack of knowledge about transmission and distribution of the disease, as described previously in Aenishaenslin et al. [35], given that the region is currently facing emergence of a new disease. Our findings may also suggest that despite their demonstrated efficacy, some preventive measures, such as applying acaricides on one’s property, are not popular in either Quebec or Switzerland, a finding which may also be explained by the low level of social acceptability for this specific measure. Previous studies have shown a low level of adoption for similar preventive measures both in low and high incidence regions for LD in other parts of the world [20,28,30,34,40].

Our findings suggest that the adoption of specific preventive behaviors also vary according to socio-demographic characteristics of respondents, such as gender, age and exposure levels and that the relationship between these characteristics and the adoption of preventive behaviors depends on the region and on the specific preventive measure. For example, being in a younger age group (either 18–34 or 35–54 *vs* 55+ year old) was positively associated with the adoption of *tick repellent* use but negatively associated with the use of *protective clothin*g. Also, a higher level of adoption was noted in women for the practice of *tick checks* in Neuchâtel but not in Montérégie, and a higher proportion was measured in women for *risk area avoidance* in Montérégie but not in Neuchâtel. In general, gender differences regarding the adoption of health-related behaviors are highly variable and depend on the type of behavior under study [41]. In Phillips and colleagues [28], women were also associated with a greater proportion of preventive behaviors including the practice of *risk area avoidance, tick checks* and *tick repellent* in residents of Nantucket Island in Massachusetts, United States. On the opposite, other studies have found no gender differences regarding LD preventive behaviors adoption [30,34].

Another main objective of this study was to test if exposure, knowledge, risk perception, and the perceived efficacy of measures were associated with the adoption of preventive behaviors to a similar degree in both regions. We calculated these associations with four multivariable logistic regression models predicting either a GPB score or an adoption score for three main specific preventive measures (OR was used as an indicator of the strength of association). We could not find significant differences between regional subsets in the strength of association when considering OR confidence intervals. On the other hand, even if we identified different significant factors between regions, we noted a good level of constancy in the strength of association, particularly in the association of risk perception and knowledge with adoption of specific measures when comparing overall models. Knowledge was also a common factor associated with preventive measures in overall models and most of the regional models, as previously reported in other regions [19,33,40]. Several studies have demonstrated that risk perception, expressed by the perceived severity of and the perceived susceptibility to LD, was associated with the adoption of preventive behaviors [19,20,24,33,34,40]. Herrington [20] compared factors associated with preventive behaviors between low and high incidence states in the United States. He observed differences in the strength of association of the perceived severity between these regions: in low incidence states, perceived severity was positively associated with the adoption of preventive behaviors (in general) while it was negatively associated with such behaviors in high-incidence states. As we used a global risk perception score (*vs* perceived severity) as an independent variable in our models, we are not able to directly compare our findings with these results.

In our study, we decided to restrict multivariable analyses to the subset of respondents who had heard about LD before the survey was administered, given that all other respondents could not have consciously applied preventive measures in order to protect themselves against LD if they did not even know about the existence of the disease. Given that a considerable number of respondents did not know about LD, especially in Montérégie where only 54% had heard about LD prior to the survey, the regional sample sizes were greatly diminished, resulting in large confidence intervals and a reduced statistical power that may partially explain the lack of differences observed in the strength of association between risk perception and the adoption of preventive behaviors between the two regions.

Nevertheless, multivariable models revealed other interesting findings. Perceived efficacy of specific preventive measures was strongly associated with the adoption of three preventive measures in our study. The perceived efficacy of a measure has previously been identified as an important predictor but the relationship was found to be stronger in our study than compared to previous studies [24,26,33].

Another interesting observation is that ‘living in the Montérégie region’ was positively associated with the use of *tick repellent* in the overall models, while it was negatively associated with the practice of *tick checks* and the use of *protective clothing*. One hypothetical explanation is that applying repellent is already well accepted by residents of the region for other reasons, such as to protect themselves against mosquitoes, and the risk of West Nile virus transmission, which is also present in this region [42]. This context is different in Neuchâtel where the use of repellent may be less common. Finally, it may seem reasonable to conclude that living in an emerging and low incidence region such as Montérégie could be negatively associated with specific preventive behaviors as the practice of *tick checks* and the use of *protective clothing* when compared to a high incidence region such as Neuchâtel.

This study has several limitations. We obtained data from a web-based survey using panels of respondents. Thus, our study was restricted to Internet users. More aspects of the representativeness of the data are discussed in Aenishaenslin et al. [35]. Also, the cross-sectional design of our study can provide useful data but cannot establish causal relationships, and thus explains our preference for the terms ‘factors associated with preventive behaviors’ in this paper, rather than ‘determinants of preventive behaviors’. A longitudinal design would be of great interest to study temporal changes in preventive behaviors in relation to evolving levels of knowledge and risk perception, particularly in the Montérégie context, where LD is emerging and where such changes will certainly be important in the coming years.

This study was carried out in two regions that were chosen based on their contrasting LD epidemiological situation. Differences observed between the two populations cannot be explained based on their LD epidemiological statuses alone. Other unmeasured contextual factors certainly have an impact on preventive behaviors, such as culture, societal values, and public health communication efforts. Our regional results should therefore be viewed as two case studies, and should be interpreted with respect to their regional specific contexts.

Another limit of the study is that several variables included in our analyses were categorized or dichotomized, resulting in a partial loss of information when compared to the raw survey data which was predominantly ordinal. This was done to allow a useful interpretation of the results in the public health context, to carry out multivariable logistic regression analyses and to maximise statistical power in our analyses.

One explanation for the high OR values found for the perceived efficacy of preventive measures in our study could be the desirability bias of the respondents. Perceived efficacy was based on survey data, and thus the assessment of the adoption of preventive behaviors was self-reported. This may introduce bias such as desirability bias in this measurement, and this bias may be exacerbated when respondents believe in the perceived efficacy of a measure. This may have increased the proportion of reported preventive behaviors and may have moved the estimate of the association between the perceived efficacy and the adoption of the measure in question away from the null. Studies focusing specifically on measuring the *observed* adoption (*vs* self-reported) of protective behaviors in relation to the perceived efficacy of a behavior may be of great interest for future research.

Finally, our study measured the association between factors with preventive behaviors, but besides the statistical significance of these factors, quantitative analysis cannot fully explain the relationships between these variables and cannot provide a deep understanding of the motivations and barriers of adoption of preventive behaviors. Qualitative studies may provide essential insights that may help deepen our understanding and ability to interpret behavior related studies, for example to explain observed differences in behavior between age categories.

## Conclusions

This study highlights the importance for public health authorities to improve their understanding and ability to monitor key social factors known to influence the adoption of proposed preventive measures in targeted populations. Improved understanding and monitoring of key social factors will help establish effective prevention programs that are well adapted to populations and their epidemiological contexts. Two key messages should be highlighted in particular from this study. First, our results suggest that a high risk perception by the population could increase the level of adoption of proposed preventive behaviors in regions where LD is emerging, an effect that could not be statistically verified in the other region where LD is endemic. Second, the perceived efficacy of a specific preventive measure seems to represent a reliable predictor for the adoption of such measures in both emerging and endemic regions. These two observations may lead to practical considerations for public health authorities with regards to the importance of the epidemiological status of a region in the overall design of prevention campaigns and to the integration of communication messages directly targeted at enhancing positive perception of selected preventive measures with the objective of increasing the efficacy of prevention efforts.

## Additional file

Additional file 1:
**Lyme disease risk knowledge, risk perceptions and behaviors questionnaire (in French).** This file presents the complete questionnaire designed and used in Quebec for this study.
