# Supplementary material for: Factors associated with preventive behaviors regarding Lyme disease in Canada and Switzerland: a comparative study
Source: BMC Public Health. 2015 Feb 25;15:185. doi: 10.1186/s12889-015-1539-2 (PMC4349712; doi:10.1186/s12889-015-1539-2)
Supplement: Additional file 1: — Lyme disease risk knowledge, risk perceptions and behaviors questionnaire (in French). This file presents the complete questionnaire designed and used in Quebec for this study. [file 12889_2015_1539_MOESM1_ESM.pdf]

Questionnaire

« Évaluation de la perception du risque de la maladie de Lyme par le grand public»

Volet québécois

Titre du projet de recherche

Perception et gestion du risque:
Étude comparative de la problématique de la maladie de Lyme au Québec et en Suisse

Chercheure-Étudiante : Cécile Aenishaenslin, DMV, MSc
Directeur : Pascal Michel, DMV, PhD (Université de Montréal)
Co-directeur : André Ravel, DMV, PhD (Université de Montréal)

Version du 10 octobre 2012

Université de Montréal

1. Vous habitez la région de la Montérégie depuis :

- ☐ Moins d'un an
- ☐ 1 à 5 ans
- ☐ 6 à 10 ans
- ☐ Plus de 10 ans

2. Vous avez entendu parler de la maladie de Lyme, aussi appelé borréliose, pour la première fois :

- ☐ Cette semaine
- ☐ Au cours du dernier mois
- ☐ Cette année, mais il y a plus d'un mois
- ☐ Il y a plus d'un an
- ☐ Je n'ai jamais entendu parler de la maladie de Lyme **(sauter les questions 3 à 6)**

Veuillez répondre aux questions 3, 4, 5 et 6 selon vos connaissances actuelles. Cochez toutes les réponses qui s'appliquent:

3. La maladie de Lyme se transmet à l'homme:

- ☐ Par contact avec des rongeurs
- ☐ Par contact avec un individu
- ☐ Par contact avec un chien infecté
- ☐ Par une piqûre de tique
- ☐ Par une piqûre de moustique
- ☐ Je ne sais pas

4. Le premier symptôme de la maladie de Lyme est généralement:

- ☐ De la diarrhée
- ☐ Des vomissements
- ☐ De la toux
- ☐ Une plaque rougeâtre sur la peau
- ☐ De la congestion nasale
- ☐ Je ne sais pas

5. Lorsqu'elle est détectée rapidement, la maladie de Lyme peut être traitée :

- ☐ Avec des antibiotiques sous forme de comprimés
- ☐ Avec des crèmes appliquées sur la peau
- ☐ Avec des médicaments contre la toux
- ☐ Il n'y a pas de traitement pour la maladie de Lyme mais un vaccin préventif existe
- ☐ Il n'y a ni traitement ni vaccin contre la maladie de Lyme
- ☐ Je ne sais pas

6. En Montérégie, il est possible de contracter la maladie de Lyme:

- ☐ Seulement dans les régions boisées situées à moins de 15km de la frontière américaine
- ☐ Dans certaines régions boisées dont des parcs d'accès public

☐ Avec les connaissances scientifiques actuelles, on ne sait pas s'il est possible de contracter la maladie de Lyme en Montérégie
☐ Il n'est pas possible de contracter la maladie de Lyme en Montérégie actuellement
☐ Je ne sais pas

Lisez maintenant le texte suivant :

La maladie de Lyme, ou borréliose, est une maladie qui se transmet par une piqûre de tique infectée par une bactérie. Les tiques qui peuvent transmettre la maladie sont présentes dans les régions boisées et les hautes herbes dans le sud-ouest du Québec dont la Montérégie. Pour survivre et transmettre la maladie à l'homme, ces tiques ont besoin des petits rongeurs sauvages comme les souris, et des cerfs de virginie (chevreuils). Le nombre de tiques dans l'environnement est donc dépendant de la présence de ces animaux dans les forêts.

Au Québec, il y a actuellement seulement quelques cas de maladie de Lyme chez l'humain par année, mais il semble que ce nombre de cas augmente progressivement. Dans la plupart des cas, cette maladie cause d'abord une plaque rouge sur la peau de la forme d'une cible, et peut causer des douleurs aux articulations et des maux de tête. Elle peut être traitée par des antibiotiques, ce qui permet généralement une guérison complète. Si elle n'est pas traitée, elle peut occasionnellement engendrer des complications et toucher le cœur, le système nerveux et les articulations, et causer des problèmes persistants.

DONNER SI POSSIBLE L'ACCES AU TEXTE POUR TOUTES LES QUESTIONS SUBSÉQUENTES.

Avec ces informations en tête, répondez maintenant aux questions suivantes :

7. Si je n'applique pas de mesures de protection particulière, le risque que j'attrape la maladie de Lyme en Montérégie est :

   ☐ Très élevé
   ☐ Plutôt élevé
   ☐ Ni élevé, ni faible
   ☐ Plutôt faible
   ☐ Très faible

8. La maladie de Lyme est une maladie très grave :

   ☐ Tout à fait d'accord
   ☐ Plutôt en accord
   ☐ Ni en accord, ni en désaccord
   ☐ Plutôt en désaccord
   ☐ Tout à fait en désaccord

9. Il serait facile pour moi de me protéger contre la maladie de Lyme:

   ☐ Tout à fait d'accord
   ☐ Plutôt en accord
   ☐ Ni en accord, ni en désaccord
   ☐ Plutôt en désaccord
   ☐ Tout à fait en désaccord

10. J'ai le sentiment qu'il existe de grandes incertitudes scientifiques sur la maladie de Lyme:

   ☐ Tout à fait d'accord
   ☐ Plutôt en accord

☐ Ni en accord, ni en désaccord
☐ Plutôt en désaccord
☐ Tout à fait en désaccord

11. Je suis inquiet par l'idée de contracter la maladie de Lyme:

☐ Tout à fait d'accord
☐ Plutôt en accord
☐ Ni en accord, ni en désaccord
☐ Plutôt en désaccord
☐ Tout à fait en désaccord

12. Sans mesures préventives, les résidents de la Montérégie, incluant les enfants, ont un risque important d'attraper la maladie de Lyme:

☐ Tout à fait d'accord
☐ Plutôt en accord
☐ Ni en accord, ni en désaccord
☐ Plutôt en désaccord
☐ Tout à fait en désaccord

Questions 13 à 20 : Acceptabilité des mesures préventives
Certaines mesures peuvent contribuer à prévenir la maladie de Lyme**. En imaginant que les mesures suivantes sont efficaces ET techniquement faisables**, veuillez choisir l'option la plus appropriée.

Q.	La mesure suivante **respecte mes valeurs et mes principes personnels :**	Tout à fait acceptable	Plutôt acceptable	Ni acceptable, ni inacceptable	Plutôt inacceptable	Tout à fait inacceptable
13	Appliquer des pesticides dans l'environnement pour diminuer la présence des tiques	☐	☐	☐	☐	☐
14	Utiliser des techniques de contrôle biologique dans l'environnement pour diminuer la présence des tiques (par exemple, mettre dans l'environnement des champignons qui peuvent réduire le nombre de tiques)	☐	☐	☐	☐	☐
15	Enlever de la végétation dans les régions boisées pour diminuer le nombre de tiques	☐	☐	☐	☐	☐
16	Protéger les chevreuils contre les tiques pour diminuer le nombre de tiques	☐	☐	☐	☐	☐
17	Contrôler le nombre de chevreuils dans les régions boisées d'accès public pour diminuer le nombre de tiques	☐	☐	☐	☐	☐
18	Empêcher les chevreuils de fréquenter les régions boisées d'accès public en mettant des barrières pour diminuer le nombre de tiques	☐	☐	☐	☐	☐
19	Protéger les petits rongeurs contre les tiques pour diminuer le nombre de tiques infectées	☐	☐	☐	☐	☐
20	Vacciner les petits rongeurs pour les protéger contre la bactérie qui cause la maladie de Lyme pour diminuer le nombre de tiques infectées	☐	☐	☐	☐	☐

Questions 21 à 40: Efficacité des mesures préventives
Pour chacune des mesures préventives suivantes, veuillez cocher l'option la plus appropriée, **sans considérer si ces mesures s'opposent ou non à vos valeurs et vos principes personnels.**

Q.	La mesure suivante **est efficace pour protéger la population** contre la maladie de Lyme:	Tout à fait d'accord	Plutôt en accord	Ni en accord, ni en désaccord	Plutôt en désaccord	Tout à fait en désaccord
21	Appliquer des pesticides dans l'environnement pour diminuer la présence des tiques	☐	☐	☐	☐	☐
22	Utiliser des techniques de contrôle biologique dans l'environnement pour diminuer la présence des tiques	☐	☐	☐	☐	☐
23	Enlever de la végétation dans les régions boisées pour diminuer le nombre de tiques	☐	☐	☐	☐	☐
24	Protéger les chevreuils contre les tiques pour diminuer le nombre de tiques	☐	☐	☐	☐	☐
25	Contrôler le nombre de chevreuils dans les régions boisées d'accès public pour diminuer le nombre de tiques	☐	☐	☐	☐	☐
26	Empêcher les chevreuils de fréquenter les régions boisées d'accès public en mettant des barrières pour diminuer le nombre de tiques	☐	☐	☐	☐	☐
27	Protéger les petits rongeurs contre les tiques pour diminuer le nombre de tiques infectées	☐	☐	☐	☐	☐
28	Vacciner les petits rongeurs pour les protéger contre la bactérie qui cause la maladie de Lyme pour diminuer le nombre de tiques infectées	☐	☐	☐	☐	☐

Q.	La mesure suivante **est efficace pour se protéger** contre la maladie de Lyme:	Tout à fait d'accord	Plutôt en accord	Ni en accord, ni en désaccord	Plutôt en désaccord	Tout à fait en désaccord
29	Chercher et enlever les tiques sur soi-même après un séjour dans une région boisée	☐	☐	☐	☐	☐
30	Porter des vêtements longs qui recouvrent les jambes jusqu'aux chaussures (par exemple, mettre son bas de pantalon dans ses chaussettes)	☐	☐	☐	☐	☐
31	Utiliser des insectifuges contenant du DEET sur la peau et/ou sur les vêtements	☐	☐	☐	☐	☐
32	Éviter les régions boisées pendant la période à risque	☐	☐	☐	☐	☐
33	Mettre des pesticides sur sa propriété	☐	☐	☐	☐	☐
34	Tondre régulièrement la pelouse sur sa propriété	☐	☐	☐	☐	☐

Q.	En Montérégie, **j'applique cette mesure** pour me protéger de la maladie de Lyme:	Toujours	Fréquem-ment[1]	Rarement[2]	Jamais	Il m'arrive d'appliquer cette mesure, mais pas pour me protéger de la maladie de Lyme	Ne s'applique pas à ma situation
35	Chercher et enlever les tiques sur soi-même après un séjour dans une région boisée	☐	☐	☐	☐	☐	☐
36	Porter des vêtements longs qui recouvrent les jambes jusqu'aux chaussures (par exemple, mettre son bas de pantalon dans ses chaussettes)	☐	☐	☐	☐	☐	☐
37	Utiliser des insectifuges contenant du DEET sur la peau et/ou sur les vêtements	☐	☐	☐	☐	☐	☐
38	Éviter les régions boisées pendant la période à risque	☐	☐	☐	☐	☐	☐
39	Mettre des pesticides sur ma propriété	☐	☐	☐	☐	☐	☐
40	Tondre régulièrement la pelouse sur ma propriété	☐	☐	☐	☐	☐	☐

[1] Habituellement, j'applique cette mesure, mais il m'arrive de ne pas le faire.
[2] Habituellement, je n'applique pas cette mesure préventive, mais je l'ai déjà fait.

41. Actuellement, je pense que le gouvernement met en place des mesures appropriées pour contrôler la maladie de Lyme :

- ☐ Tout à fait d'accord
- ☐ Plutôt en accord
- ☐ Ni en accord, ni en désaccord
- ☐ Plutôt en désaccord
- ☐ Tout à fait en désaccord

42. Lorsque le gouvernement doit décider des mesures à mettre en place pour prévenir la maladie de Lyme, plusieurs enjeux sont à considérer.
Selon vos principes et valeurs, mettez en ordre d'importance les enjeux suivants (1 à 5, **1 étant le plus important et 5, le moins important**). En cas d'égalité de deux enjeux, indiquez deux fois le même numéro :

Rang (1à 5)	Description de l'enjeu
	Protéger la santé des êtres humains.
	Protéger la santé de la faune sauvage.
	Protéger la qualité de l'environnement, incluant l'air, l'eau, les végétaux et les sols.
	Respecter les intérêts et les valeurs des citoyens.
	Minimiser les dépenses publiques.

43. Concernant l'environnement extérieur de votre résidence personnelle, quel est l'énoncé qui s'applique le mieux à votre situation :

☐ Je n'ai pas accès à une cour extérieure
☐ J'ai accès à une cour extérieure mais je n'ai pas la responsabilité de son entretien
☐ J'ai accès à une cour extérieure et j'ai la responsabilité de son entretien

44. Vous visitez les régions boisées entre mai et octobre dans l'une ou plusieurs des régions suivantes : en Montérégie ou en Estrie au Québec ou dans le Nord-est des États-Unis :

☐ Plus de 25 fois par année
☐ De 11 à 25 fois par année
☐ De 2 à 10 fois par année
☐ Moins de 2 fois par année
☐ Jamais

45. Avez-vous un chien actuellement?

☐ Oui
☐ Non

46. Est-ce que vous croyez avoir déjà eu la maladie de Lyme?

☐ Oui
☐ Non

47. Connaissez-vous personnellement quelqu'un qui a déjà eu la maladie de Lyme?

- ☐ Oui
- ☐ Non

48. Vous êtes :

- ☐ Une femme
- ☐ Un homme

49. Dans quelle catégorie d'âge vous situez-vous?

- ☐ 18-24 ans
- ☐ 25-34 ans
- ☐ 35-44 ans
- ☐ 45-54 ans
- ☐ 55-64 ans
- ☐ 65-74 ans
- ☐ 75 ans ou plus
- ☐ Je préfère ne pas répondre

50. Quel est le revenu total avant impôt de tous les membres du foyer pour l'année 2011 ?

- ☐ 19 999$ ou moins
- ☐ Entre 20 000 et 39 999$
- ☐ Entre 40 000 et 59 999$
- ☐ Entre 60 000 et 79 999$
- ☐ Entre 80 000 et 99 999
- ☐ Entre 100 000$ et 119 999$
- ☐ 120 000$ ou plus
- ☐ Je préfère ne pas répondre

51. Quel est le niveau de scolarité le plus haut que vous avez complété?

- ☐ Primaire (7 ans ou moins)
- ☐ Secondaire (DES de formation générale ou professionnelles (8 à 12 ans))
- ☐ Collégial (DEC de formation préuniversiaires, de formation technique, certificats (CEP), attestations (AEC) ou diplôme de perfectionnement (DEP))
- ☐ Universitaire : certificats et diplômes
- ☐ Universitaire : 1er cycle Baccalauréat
- ☐ Universitaire : 2ième cycle Maîtrise
- ☐ Universitaire : 3ième cycle Doctorat
- ☐ Autre : ____

52. Veuillez saisir les 6 lettres de votre code postal :

Merci !

Pour obtenir plus d’informations : communiquer avec le service téléphonique Info-Santé au 8-1-1 ou visiter le site Web du ministère de la Santé et des Services sociaux portant sur la maladie de Lyme <http://www.msss.gouv.qc.ca/sujets/santepub/maladie-lyme.php>.
